# Efficacy of ibuprofen on prevention of high altitude headache: A systematic review and meta-analysis

**DOI:** 10.1371/journal.pone.0179788

**Published:** 2017-06-20

**Authors:** Juan Xiong, Hui Lu, Rong Wang, Zhengping Jia

**Affiliations:** Key Laboratory of the plateau of the environmental damage control, Lanzhou General Hospital of Lanzhou Military Command, Lanzhou, China; Cardiff University, UNITED KINGDOM

## Abstract

**Objective:**

Ibuprofen is used to prevent high altitude headache (HAH) but its efficacy remains controversial. We conducted a systematic review and meta-analysis of randomized, placebo-controlled trials (RCTs) of ibuprofen for the prevention of HAH.

**Methods:**

Studies reporting efficacy of ibuprofen for prevention of HAH were identified by searching electronic databases (until December 2016). The primary outcome was the difference in incidence of HAH between ibuprofen and placebo groups. Risk ratios (RR) were aggregated using a Mantel-Haenszel random effect model. Heterogeneity of included trials was assessed using the *I*^*2*^ statistics.

**Results:**

In three randomized-controlled clinical trials involving 407 subjects, HAH occurred in 101 of 239 subjects (42%) who received ibuprofen and 96 of 168 (57%) who received placebo (RR = 0.79, 95% CI 0.66 to 0.96, Z = 2.43, P = 0.02, *I*^*2*^ = 0%). The absolute risk reduction (ARR) was 15%. Number needed to treat (NNT) to prevent HAH was 7. Similarly, The incidence of severe HAH was significant in the two groups (RR = 0.40, 95% CI 0.17 to 0.93, Z = 2.14, P = 0.03, *I*^*2*^ = 0%). Severe HAH occurred in 3% treated with ibuprofen and 10% with placebo. The ARR was 8%. NNT to prevent severe HAH was 13. Headache severity using a visual analogue scale was not different between ibuprofen and placebo. Similarly, the difference between the two groups in the change in SpO_2_ from baseline to altitude was not different. One included RCT reported one participant with black stools and three participants with stomach pain in the ibuprofen group, while seven participants reported stomach pain in the placebo group.

**Conclusions:**

Based on a limited number of studies ibuprofen seems efficacious for the prevention of HAH and may therefore represent an alternative for preventing HAH with acetazolamide or dexamethasone.

## Introduction

Acute mountain sickness (AMS) results from hypoxemia and is marked by a group of specific symptoms including headache, nausea, weakness, dizziness, and sleep disturbances [1]. AMS occurs frequently in non-altitude-acclimatized individuals upon acute exposure to hypobaric hypoxia, generally above 2500 m [1]. High altitude headache (HAH) may occur in isolation or as a sentinel symptom in developing AMS and is a necessary criterion for the diagnosis of AMS using the Lake Louise Score (LLS) system [2]. Although HAH and AMS are usually self-limiting, if not treated properly, they may progress to life-threatening high altitude cerebral edema (HACE) [1].

Scarce evidence exists for the effects of pharmacological or other prevention strategies for HAH. Although high-quality evidence is lacking, generally, the most efficient method of prophylaxis against HAH is by ascending gradually to allow enough time for altitude acclimatization [1, 3]. If fast ascent is inevitable, the use of drug prophylaxis a few hours before going up to high altitude is useful for prevention of HAH [4–7]. Although acetazolamide has been employed as the mainstay medicine for prevention of HAH and AMS for decades [8, 9], multiple studies suggested that non-steroidal anti-inflammatory drugs (NSAIDs) may also guard against HAH and, possibly, AMS [4–6, 10–14]. Ibuprofen, an NSAID, via cyclo-oxygenases inhibition counters the production of prostaglandins and the inflammatory cascade, thus limiting chemical irritants known to sensitize meningo-vascular receptors that mediate nociception [15]. Whether ibuprofen might mask the cardinal HAH symptom of AMS without preventing AMS or prevent AMS and accelerate high-altitude acclimatization, is still controversial [15].

There are only few studies that looked at the efficacy of ibuprofen in the prevention of HAH. They are limited by their small sample size and limited methodological quality. However, there is little critically appraised evidence, such as systematic reviews or meta-analyses, on the potential benefits of ibuprofen on HAH. One recent systemic review and meta-analysis suggested that NSAIDs might be an alternative for AMS pharmacological prevention [16]. Systematic meta-analysis of the efficacy of ibuprofen for preventing HAH remained to be done. We therefore collected the available evidence and reviewed it systematically for the efficiency of ibuprofen for the prevention of HAH. Since assessment of HAH is subjective and potentially prone to bias, we settled to include only randomized and placebo-controlled studies which clearly defined the diagnosis of HAH.

## Methods

Trials assessing efficacy of ibuprofen for the prophylaxis of HAH controlled with placebo were considered for inclusion in our present systematic review. We followed the preferred reporting items for systematic reviews and meta-analyses consensus statement for reporting this systematic review.

### Database and search strategy

A comprehensive literature search (last update, December 2016) was carried out in PubMed, Web of Science, SCOPUS, the Cochrane Library, Google Scholar, and China National Knowledge Infrastructure (CNKI) for full texts of randomized trials that compared ibuprofen with placebo for the prevention of HAH. Terms that were used in searching included ‘advil’, ‘andran’, ‘brufen’, ‘bufedon’, ‘dignoflex’, ‘emodin’, ‘fenbid’, ‘ibuflam’, ‘ibumetin’, ‘ibupan’, ‘ibuprofen’, ‘ibuprophen’, ‘lopane’, ‘melfen’, ‘menadol’, ‘motrin’, ‘novogent’, ‘pedibu’, ‘perofen’, ‘proflex’, ‘rupan’, ‘spedifen’ in combination with ‘high altitude illness’, “acute high altitude illness”, ‘‘acute mountain sickness”, or ‘high altitude headache.”

### Selection of studies

One author screened the abstracts of all retrieved titles and decided whether the paper contained potentially relevant data. And this was checked by a second author. We selected studies that were randomized and compared the use of ibuprofen versus placebo for the prevention of HAH. The eligibility criteria for this meta-analysis were 1) randomized controlled trials (RCTs) of healthy human subjects between age 18 and 60 years, 2) prophylaxis with either ibuprofen or placebo for HAH, 3) HAH was clearly defined and quantified on a Likert scale such as in the LLS System, and 4) performed in subjects who were ascending to high altitude by climbing or transport or simulated in hypobaric chambers. The RCTs were excluded if they 1) were unrelated to the current research topic of this systematic review, 2) did not primarily assess prevention of HAH, 3) did not show incidence or numbers of HAH subjects, 4) included subjects that were pregnant, 5) included participants had symptoms consistent with AMS at baseline, and 6) included subjects using any NSAIDs, steroids or acetazolamide within 1 week before enrolment. Nonrandomized or quasi-randomized trials, or data from abstracts or letters were not taken into consideration.

### Data abstraction

We only included studies diagnosing HAH with the Lake Louise Criteria, a widely used measurement tool for diagnosing HAH. According to this scale, headache was determined as score greater than or equal to 1. Severe headache was determined by a score greater than or equal to 2 in two included trials [5, 13]. The severity of HAH was also quantified with visual analog scale (VAS) in all three included trials [5, 6, 13]. We only extracted data when they were available in dichotomous form. When HAH was reported at different time points, we extracted the highest cumulative incidence, with ibuprofen and placebo.

### Assessment of methodological quality

Potentially relevant reports were read by all authors independently. The quality of included studies was independently evaluated by two reviewers using the guidelines provided by Cochrane Collaboration tool for assessing risk of bias. All of the studies were assessed for random sequence generation, allocation concealment, blinding of participants, incomplete outcome data, selective outcome reporting, and free from other biases. Each domain of risk was assigned as ‘adequate’ for low risk, ‘inadequate’ for high risk, and unclear in cases not mentioned.

### Outcome measures

The primary outcome measure for this systematic review was prevention of HAH (i.e. a score of 0 for HAH with the LLS). Studies without a clear assessment of HAH were excluded. Secondary outcomes included incidence of severe HAH, difference in arterial oxygen saturation (SpO_2_), and the severity of headache on VAS.

### Statistical analysis

We analyzed dichotomous data by calculating risk ratios with 95% confidence intervals. We used a Mantel-Haenszel random effect model to aggregate data. Heterogeneity between studies was tested using the Cochrane Q and *I*^*2*^ statistics. Heterogeneity of included trials was interpreted by *I*^*2*^ values. *I*^*2*^ values less than 30% were defined as low heterogeneity; less than 60% were considered as moderate heterogeneity; and more than 60% were determined as high heterogeneity [17]. A α level of 0.05 was used as the level of significance. The results are reported in a forest plot with 95% CI. RRs were calculated for each dichotomous outcome and standard mean difference for continuous outcomes. Number needed to treat (NNT) to prevent one event of HAH was determined by the following formula: NNT = 1/ (CER-EER) (CER is control event risk; EER is experimental event risk). We assessed publication bias using a funnel plot and Egger’s test for asymmetry. Revman 5.0 software provided by the Cochrane Collaboration was used for data processing and analysis.

## Results

### Screening results and characteristics of included studies

**Fig 1:**
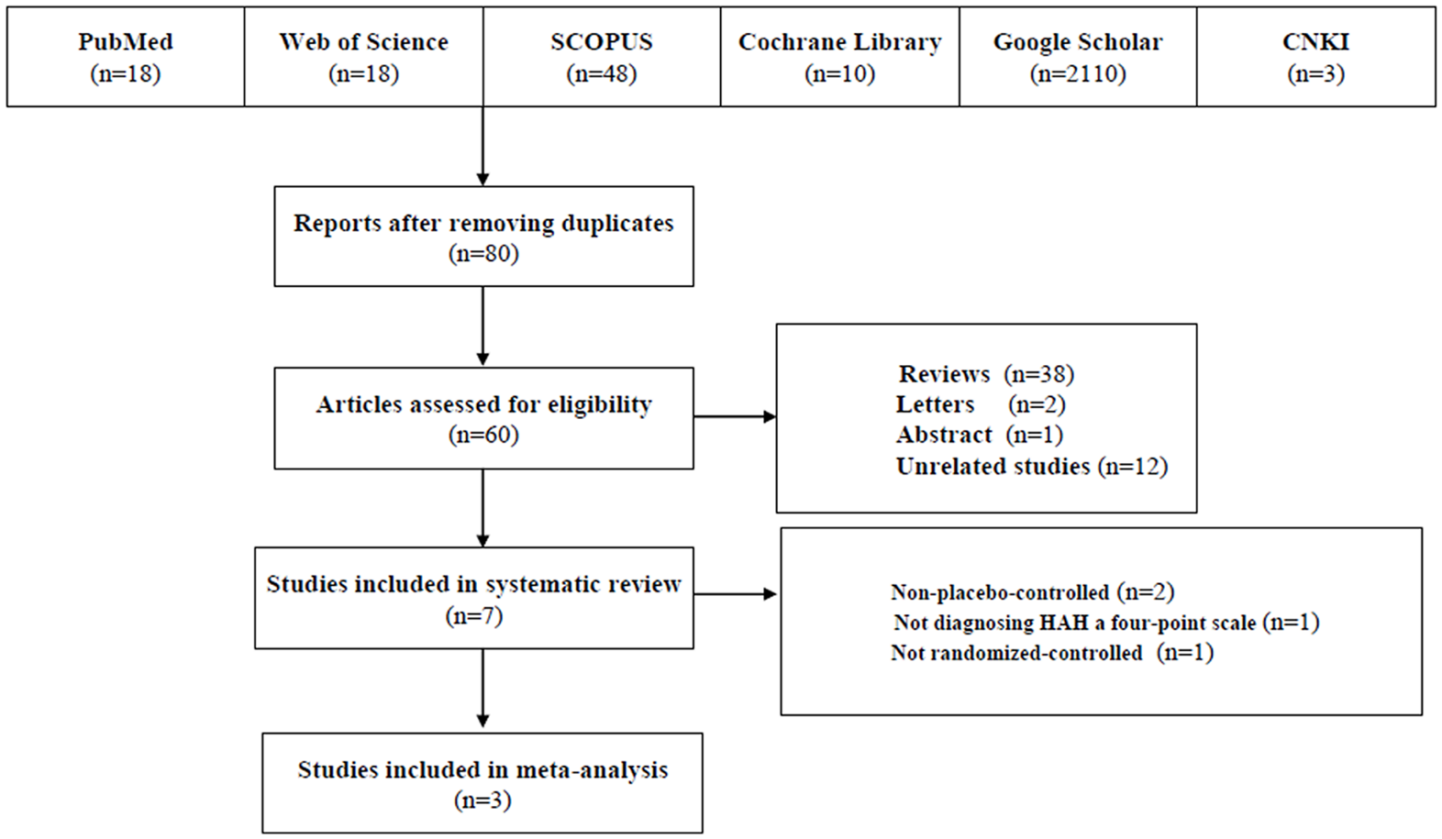
Flow of the results of literature search, review, and selection.

The result of the search strategy is summarized in Fig 1. We retrieved 2207 articles of which 3 RCTs were eventually included in our analyses [5, 6, 13]. Studies were included because they met the inclusion criteria and were therefore all randomized and placebo-controlled trials for the prevention of HAH with ibuprofen. The included trials were published between 2010 and 2012. Data from 407 subjects were analyzed; 239 subjects received ibuprofen; 168 subjects were controls with placebo. The three included studies used the Lake Louise Criteria for diagnosing HAH [5, 6, 13]. All included trials used a four-point Likert scale (0 = none, 1 = mild, 2 = moderate, 3 = severe) for diagnosing HAH. The characteristics of the included studies are listed in Table 1. Four clinical trials with ibuprofen were excluded because they were non-placebo-controlled [11, 14], not diagnosing HAH by either a four-point scale or VAS [10], and not being randomized-controlled [18]. Another clinical study with ibuprofen was not included in our present meta-analysis as this study focused on the hypoxic ventilatory response (HVR) rather than HAH [19].

**Table 1:** Characteristics of included studies.

Reference	Location	Number of subjects		Intervention profile	End point assessment	Ascent mode	Ascent range (m)
		Ibuprofen	Placebo				
Gertsch et al., 2010	Himalayas, Nepali	103	65	600 mg of ibuprofen three times daily	Four-point scale of diagnosis of HAH with VAS	Climbing	4280/4358–4928
Gertsch et al., 2012	Himalayas, Nepali	110	73	600 mg of ibuprofen three times daily	Four-point scale of diagnosis of HAH with VAS	Climbing	4280/4358–4928
Lipman et al., 2012	White Mountains, California	44	42	600 mg of ibuprofen three times daily	Four-point scale of diagnosis of HAH with VAS	Transport and Climbing	1240–3810

The oral administration profile of ibuprofen used before the ascent to high altitudes varied among the three included studies. The two studies of Gertsch and others used 600 mg of ibuprofen three times daily before the ascent [5, 13]. Lipman and his colleagues started ibuprofen 3 times daily on the day of ascent with the first dose of ibuprofen at the baseline altitude of 1240m, the second dose during ascent, and the third dose at destination (3810m) [6].

The two trials by Gertsch et al. focused on ascent from an altitude of 4280m or 4358m to 4928m by climbing [5, 13] while the Lipman et al. trial studied ascent from 1240m to 3810m by transportation and climbing within 12 hours [6]. Only one study mentioned the altitude of permanent residence of subjects [6]. No study mentioned the body mass index (BMI) of participants. The three included trials showed no significant difference in age of participants between the ibuprofen and placebo groups (P = 0.48).

### Methodological quality of included trials

The three included trials generally were of good methodological quality according to the predefined quality assessment criteria. Randomization and blinding was mentioned in all trials. Allocation concealment and blinding of outcome assessment were also mentioned. All trials reported incomplete or selective outcome data. All trials were free from other biases.

### Prevention of HAH in all included RCTs

**Fig 2:**
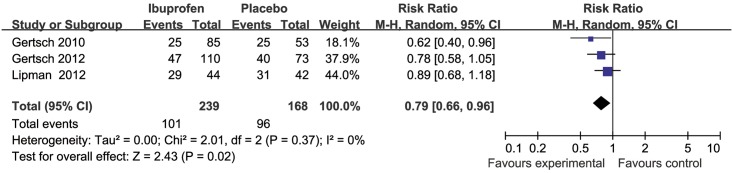
Forest plot of weighted mean difference in incidence of HAH in placebo and ibuprofen groups for all clinical trials.

HAH occurred in 101 of 239 (42%) treated with ibuprofen and in 96 of 168 (57%) with placebo. Compared with placebo and independent of the baseline risk ratio (RR), the combined RR of developing HAH with ibuprofen was 0.79 (95% CI 0.66 to 0.96, Z = 2.43, P = 0.02, *I*^*2*^ = 0%) (Fig 2). The absolute risk reduction (ARR) was 15%. The NNT to prevent HAH was 7.

**Fig 3:**
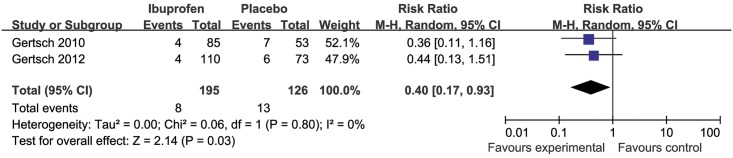
Forest plot of weighted mean difference in incidence of severe HAH in placebo and ibuprofen groups for all clinical trials.

Compared with placebo and independent of the baseline RR, the combined RR of developing severe HAH with ibuprofen compared with placebo and independent of the baseline RR was 0.40 (95% CI 0.17 to 0.93, Z = 2.14, P = 0.03, *I*^*2*^ = 0%) (Fig 3). Severe HAH occurred in 8 of 195 (3%) treated with ibuprofen and 13 of 126 (10%) with placebo. The ARR was 8%. The NNT to prevent severe HAH was 13.

**Fig 4:**
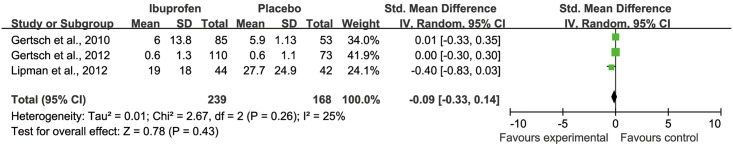
Forest plot of weighted mean difference in severity of HAH using VAS in placebo and ibuprofen groups.

Minor outcome of headache severity using VAS was not different between ibuprofen and placebo (inverse variance of standard mean difference (IV) = -0.09; 95% CI -0.33 to 0.14, Z = 0.78, p = 0.43, *I*^*2*^ = 25%) (Fig 4). Minor outcome decrease of SpO_2_ from baseline was not different between ibuprofen and placebo (IV = 0.01; 95% CI -0.31 to 0.33, Z = 0.06, p = 0.95, *I*^*2*^ = 59%).

### Adverse effects

Two included trials with ibuprofen reported that no adverse effects were observed using ibuprofen for prevention of HAH [6, 13]. One included RCT with ibuprofen reported one participant with black stools and three participants with stomach pain in the ibuprofen group, while seven participants reported stomach pain in the placebo group [5].

### Publication bias

**Fig 5:**
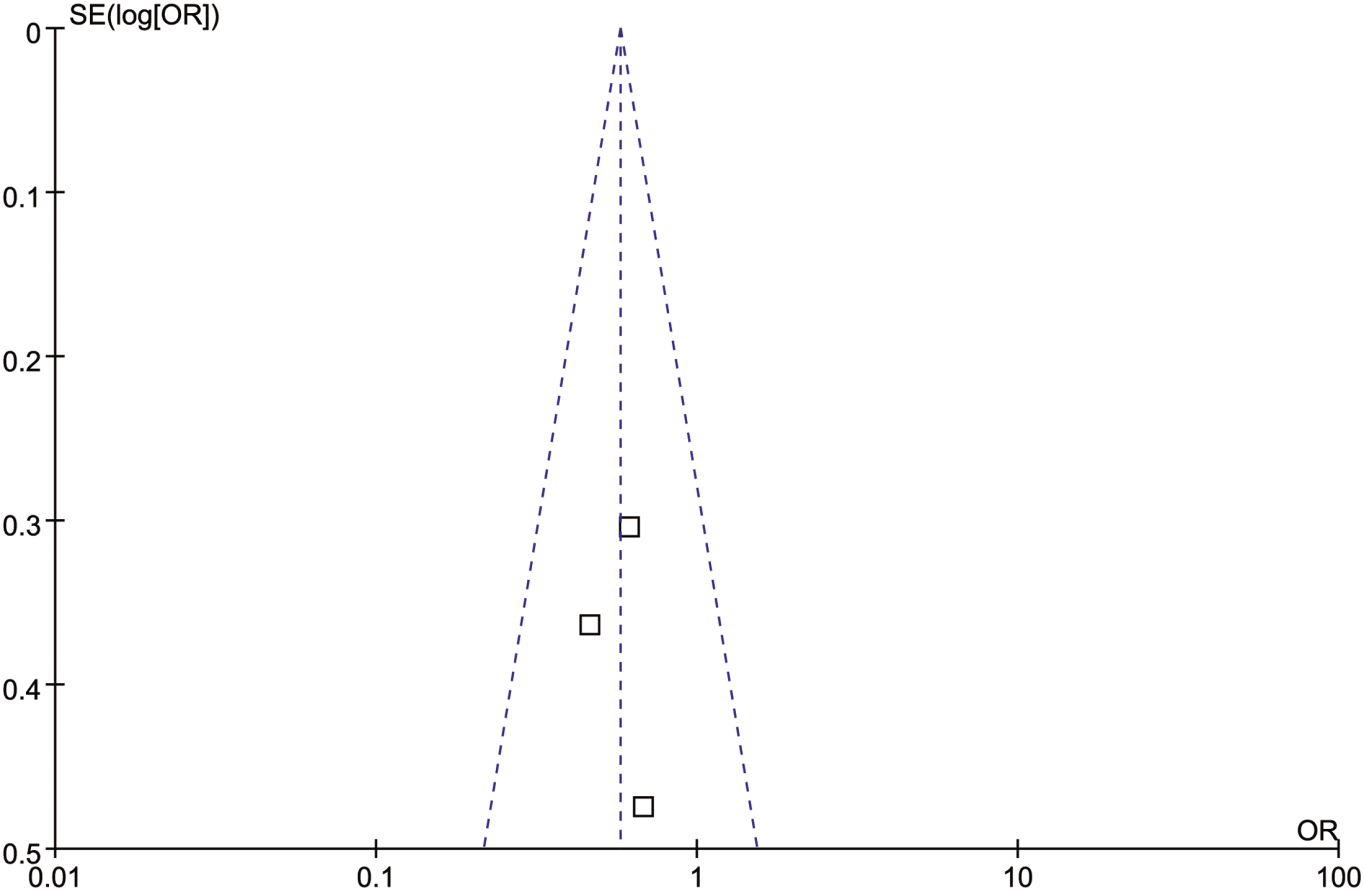
Funnel plot derived from all included clinical trials selected for meta-analysis.

Begg’s funnel plot was used for the assessment of the publication bias of selected studies used for meta-analysis. Result showed no obvious asymmetry, indicating no publication bias was detected (Fig 5).

## Discussion

In this systematic review, we summarize the three valid RCTs to date reporting ibuprofen for prophylaxis of HAH. Analyzed together these studies suggest that ibuprofen is efficacious for the prevention of HAH (Figs 2 and 3). However, ibuprofen did not lead to a reduction of VAS quantified severity of HAH in those who developed a headache (Fig 4). We could further show that ibuprofen has no effect on the SpO_2_ reduction from baseline induced by the gain in altitude.

Our systematic review thus suggests efficacy of ibuprofen for the prevention of HAH with a RR of 0.79 (Fig 2). The efficacy of ibuprofen for the prevention of severe HAH was also significant (RR = 0.40). One randomized-controlled headache evaluation at altitude trial comparing ibuprofen to acetazolamide showed that ibuprofen and acetazolamide have similar efficacy in preventing HAH and both were more effective than placebo [14]. One older randomized-controlled within-patient crossover trial compared to placebo suggested that ibuprofen could dramatically reduce HAH and HAH relief time [10]. One non-randomized-controlled trial demonstrated that ibuprofen can reduce the frequency and severity of HAH [18]. Our results are not consistent with one recent meta-analysis with NSAIDs for prevention of AMS, which suggested ibuprofen has a significant effect on headache severity using VAS [15].

Our finding that ibuprofen did not reduce VAS-quantified severity of HAH in those who developed a headache despite prophylaxis (Fig 4) may suggest that ibuprofen’s preventive effect does not relate to its analgesic properties. The exact mechanism of developing HAH remains elusive. This knowledge gap about the etiology of HAH frustrates to reveal the actual mechanisms of ibuprofen prevention of HAH. It is believed that hypoxia induced a series neurohumoral and hemodynamic responses, including an inflammatory response, disruption of the blood-brain barrier (BBB), occurrence of (sub-clinical) cerebral edema, nitric oxide-mediated activation of the trigeminovascular system, and/or inadequate venous drainage capacity [2, 20–23]. All three included studies refer to the inflammatory process being important for the pathophysiology of HAH. Ibuprofen is thought to be of benefit in preventing HAH via cyclo-oxygenases (COXs) inhibition preventing the production of prostaglandins (PGI) and the inflammatory cascade, limiting chemical irritation of meningovascular receptors that mediate nociception [24]. In agreement two randomized controlled trials suggested that aspirin is useful for preventing HAH [4, 12]. By contrast, naproxen [25] and carbasalate [26] did not prevent AMS compared with placebo. Unfortunately, both studies did not report the incidence of HAH. It is of note that paracetamol and ibuprofen have similar efficacy in preventing AMS [14]. This is of interest since paracetamol has no anti-inflammatory effects, but may prevent headache through its analgesic properties. There was no significant difference in SpO_2_ reduction from baseline between placebo and ibuprofen groups. This indirectly supports the contention that ibuprofen prevents HAH through modulation of the inflammatory process rather than through increasing SpO_2_. Whether ibuprofen speeds high altitude acclimatization or just masks symptoms of AMS by reducing HAH is still an open question.

The present mainstay drugs for the prevention of HAH and AMS are acetazolamide and dexamethasone. But can have adverse effects. Acetazolamide’s adverse effects of nausea, vertigo, and fatigue are usually well tolerated, but can be equally debilitating as AMS [26, 27]. Dexamethasone used for AMS prevention is limited because it has been linked to hyperglycemia, adrenal suppression, delirium, depression, insomnia, and mania [7, 28–31]. Also ibuprofen can have side-effects such as gastro-intestinal (GI) upset and kidney injury symptoms [32]. There is some evidence that hypoxia in combination with NSAIDs may lead to an increased risk of GI bleeding [33].

## Limitations

Our systematic review has several limitations. Firstly, good RCTs are paucity. In our present meta-analysis, only three RCTs are included. In the included trials, high proportion of patients broken the protocol or were lost to follow up. These points undoubtedly undermine the methodological quality of included trials and conclusions of our present meat-analysis. Therefore, these results of our meta-analysis should be interpreted with caution as limited number and poor methodological quality of included trials. Some non-RCTs studies suggested that ibuprofen have effects on the prevention of HAH. One randomized-controlled trial compared ibuprofen to acetazolamide showed that ibuprofen and acetazolamide have similar function on preventing HAH [14]. One older randomized-controlled within-patient crossover trial compared to placebo suggested that ibuprofen can dramatically reduce HAH and HAH relief time [10]. One non-randomized-controlled trial demonstrated that ibuprofen can reduce the frequency and severity of HAH [28]. More high quality RCTs are needed to confirm the efficacy of ibuprofen in preventing HAH. Secondly, two of the three studies included participants whom were already partly acclimatized and excluded altitude sensitive subjects (who were already sick at the inclusion site (4280m, or 4358m) [5, 6, 13]. Future trials should begin at low altitude. Similarly, further research should standardize the method of climbing to avoid bias from rate of exposure and physical exertion on the incidence of HAH. Finally, the International Headache Society (IHS) has included a definition of HAH that HAH has at least two of the following characteristics after ascent to altitude above 2500m: 1) bilateral, 2) frontal, 3) dull or pressing quality, 4) mild or moderate intensity, 5) aggravated by exertion, movement, straining, coughing, or bending. HAH develops within 24 hours after ascent and resolves within 8 hours of descent. Although we included only those studies that clearly defined HAH using a four-point scale for diagnosis of HAH, the four-point scale for diagnosis of HAH itself is prone to subjective bias.

## Conclusions

In conclusion, our systematic review suggests evidence of a benefit of prophylactic ibuprofen in the prevention of HAH. Ibuprofen may thus represent an alternative for acetazolamide or dexamethasone when taken for HAH prophylaxis. Whether ibuprofen also represents an alternative prophylaxis for AMS is still an open question.

## Supporting information

S1 Table PRISMA 2009 checklist. (DOC)
